# Neuroethics: the pursuit of transforming medical ethics in scientific ethics

**DOI:** 10.1186/s40659-016-0070-y

**Published:** 2016-02-20

**Authors:** Gustavo Figueroa

**Affiliations:** Departamento de Psiquiatría, Escuela de Medicina, Universidad de Valparaíso, Valparaíso, Chile

**Keywords:** Neuroethics, Free will, Mind-body problem, Neuroscience

## Abstract

Ethical problems resulting from brain research have given rise to a new discipline termed neuroethics, representing a new kind of knowledge capable of discovering the neural basis for universal ethics. The article (1) tries to evaluate the contributions of neuroethics to medical ethics and its suitability to outline the foundations of universal ethics, (2) critically analyses the process of founding this universal ethic. The potential benefits of applying neuroimaging, psychopharmacology and neurotechnology have to be carefully weighed against their potential harm. In view of these questions, an intensive dialogue between neuroscience and the humanities is more necessary than ever.

## Background

Toulmin provocatively postulated that medicine saved Western ethics from its implicit, although increasingly decadence product of academic discussions with little concrete value for the lives of human beings, when creating bioethics based on the urgency of physicians at the bedsides of their patients [1]. Despite his astute reflection, he did not consider two aspects. Firstly, beginning in the 1960s, philosophy made an important ethical shift as a result of a “rehabilitation of practical philosophy”, which means a priority on the practical, immediate and factual [2, 3]. On the other hand, medical ethics has a long history that it has never abjured; moreover, it has always constituted the first foundation of medicine’s ends, theories and practices, and continues to be in full force significantly in the twentieth century [4–6].

The present work has three objectives. The first is to outline the birth of neuroethics from the ethical tradition of medicine. The second is to consider the main achievements, advances and future perspectives of neuroethics, and the third is to discuss the foundations underlying this new way of understanding medical morals.

## Origin of neuroethics

Medicine emerged in Greece as a profession, that is, a *professio*, which has a religious origin: to profess is an act that demands delivering, an activity that demands committing one’s self entirely and for life. It is a kind of consecration and those who exercise it are consecrates [7, 8]. From this the Hippocratic “Oath” was born at the dawning of medicine (*tekhné iatriké*) around the fifth century B.C. and consequently every physician is committed not only to executing his/her techniques well, but also to profess a moral. This moral is not just any, but rather is one that tends toward perfection or excellence (*areté*) and the doctor is a special person because he/she seeks to conduct a virtuous life. The moral perspective has accompanied us continuously throughout history, in completely distinct civilizations like the Hindu, Jewish, Arabic and Chinese. This means that the raison d’être of the moral perspective is so deep and so deeply rooted in our tradition that any person that takes up its exercise is required to begin with a strict and solemn ritual, that of taking an oath, real or symbolic, of respect and obedience [9–11].

The situation changed dramatically after the first half of the twentieth century and in a short time bioethics burst forth, imposed itself and spread with unstoppable force; despite the numerous investigations dedicated to its genesis—medical, legal, economic, historical, philosophical-, there has not yet been a satisfactory elucidation of the motives that provoked this revolution that definitively upset the way in which medicine is practiced. In short, there is a before and an after of this event [12–14]. With a *bilocated birth*, ecological bioethics, headed by Van Rensselaer Potter [15] and medical bioethics, guided by André Hellegers and Daniel Callahan [16, 17], followed the proposal of the cancer specialist Madison: “As a new discipline….combines biological knowledge with knowledge of the systems of human values” [18]. Its objective was ambitious, to bridge two modes of understanding the condition of human illness based on biological sciences and the humanities and its values. Its spectacular expansion exceeded any prediction and in a few years it compromised not only medicine in its totality, but also law, economy, philosophy and politics. Three attributes characterize its growth when applied to human illness: to elaborate specific procedures that serve to guide medical action in its very diverse fields; a particular concern about its application with the aim that it not remain in dead letters, because of which it was necessary to recommend sanctions in cases of negligence or abuse; and developing certain principles sufficiently general that they can serve as the basis for ordering behavior and taken in account requires acceptance by all members in order to aspire to universal in an axiological and polytheistic society like that of today [19]. In other words, foundations, systems of prescriptions or procedural guides and regulated and effective sanctions.

Despite the coincidence in time, the situation was very different from the ethical problems resulting from the biotechnological revolution: uncertainty, risk and danger. In the early 1970s, the revolutionary cellular and animal virus research began to show its misty and ominous face: the growing threat to which researchers were exposing the entirety of humanity. Alarms went off vividly, recalling the words of Oppenheimer after the fateful nuclear tests, “physicists have known sin and this is a knowledge which they cannot lose” [20]. It was in June 1973 and again from February 24–27 in 1975 at the Asilomar conferences on the risks of recombinant DNA that, after heated discussion, safety guidelines were approved with two types of protective barriers, biological and physical, and four levels of risk. With what was termed the “precautionary principle” and the certain awareness that the manipulation of genetic material is always done in the context of uncertainty, GenEthics was born. Its main conclusion, unprecedented as it had never been expressed before so unequivocally, was that humanity had to be considered as a limiting end to scientific and technical interventions, for example, to positive and negative genetic engineering [21]. But at the same time the biological sciences passionately took up again the doctrine that has moved it with growing force in the beginning of the 19th century, the essence of human beings lies in their primarily biological condition and empirical data is proving that it is the gene where the ultimate truth lies.

The situation changed and again explosively when in May 2002, 150 biologists, neuroscientists, physicians, lawyers, psychologists, and sociologists met in San Francisco and proclaimed, in the words of the journalist and organizer William Safire, that neuroethics had been born and that it was characterized as “the study of ethical, legal and social questions that emerge when scientific discoveries about the brain led to medical practices, legal interpretations and health and social policies” [22]. It was concluded that neuroscience and its technology had progressed with such vigor that encompass, drive, configure and determine decisively the different areas of human activity—art, philosophy, law, economics, theology, medicine. A century ago Husserl firmly maintained that “there is no idea more powerful and whose advance is more irresistible than that of science …, nothing can stop its triumphal march” [23]; now neuroethics reaffirms it with propriety and feels authorized to assert that it is empirical science that can and should provide the fundamental responses and basic truths about the place of humans in the cosmos. In this it is similar to GenEthics, but from there also differences emerge. While GenEthics reminds us of the potential dangers, it promotes the regulation of its actions and elaborates strict precautionary protocols (which have been becoming progressively more flexible over time), Neuroethics raises as an inalienable right to investigate without limits or hindrance, has as a goal to provide the scientific basis of the ethic supported in empirical findings and assumes among its tasks that of modifying the human essence, or some its features, according to advances in research. Neurophilosophy emerged with unusual speed as a result of its proposals and in consonance with them burst forth neurophilosophy, neurotheology, neuropolitics, neuroeconomics, neuroaesthetics, neuroeducation, just to name the main disciplines, novice disciplines that have placed traditions in check and obliged them to rethink under threat of being relegated to the past.

## The neuroethics project

In 2002 Roskies proposed dividing the field into two branches that, although intimately connected, are distinct and require being differentiated in order to refine the project [24]: ethics of neuroscience, a moral framework aimed at regulating, ordering and guiding behavior in neuroscientific research and the application of knowledge with human beings, given that studies can compromise transcendental aspects of our condition. On the contrary, the neuroscience of ethics seeks to determine where morality is born as such and what is its primary substance, constituting the nucleus of the human subject and perhaps what distinguishes us from all the other beings on the planet. In other words, applied neuroethics and fundamental neuroethics, both equally products of current neuroscience [25]. While the first includes studies that generally deal with circumspect aspects, the second is more ambitious and is related to all of Western thought: it seeks to respond to the basic dimensions of our being, like the mind-body relationship, aporia determinism-freedom, self-identity, the possibility of a universal ethic, the nature of morality, the human essence as such. If we remember that for GenEthics the truth of the human being is found in the gene, now it is said to be in the neuron and consequently terms are used like “neuronal man”, “the ethical brain”, “the thinking brain”, “the computational mind”, “the empathic brain”, “synaptic self” without “philosophical zombies” [26–31].

The neuroethic project must be rightly understood. It has been developed according to hypotheses, and these, as is characteristic of the natural sciences, are generated by testing and refuting and, as a multidisciplinary work, requires close collaboration of different professionals, a task that involves stimulation, confusion and disputes. But one thing remains clears: finally one feels to be on the sure path of strict and rigorous science to understand morality, the “*Faktum* of the moral conscience”, as Kant said, although in the opposite direction of the thinker of Könisberg because it is based on sensitive empirical data [32]. The French neurophysiologist Changeux expressed with the conviction and security of a scientist when confronting a philosopher, in this case Ricoeur, on dictating the last foundation: the “natural foundation, which for me represents something without any reference to anything occult, supernatural or magical, but rather only to a material nature, *a unique and sufficient reality*, that exists and is understood only by itself” [33]. Evers called this basic position of neuroscience “illustrated materialism” [34], a posture that has come to be so penetrating that this has been termed “the century of the brain”. Despite the undeniable novelty, we must remember that it has a long tradition. “I will consider the actions and appetites of men just as if it were a question of lines, planes and bodies”, wrote Spinoza in his *Ethics* [35], and Descartes, despite the todays misunderstandings of his position, also admitted “that [man] is not of a different nature from that of fires in inanimate bodies” [36]. Moreover, this comprehension reaches even to the pre-Socratic philosophers like Democritus: “we know nothing accurately in reality but only as it changes according to the bodily condition, and the constitution of those things that flow upon the body [including the brain] and impinge upon it” [37].

## Ethics of neurocience

What areas have provided the most convincing answers in this field? This is not easy to assess because the perspectives, questions and techniques of execution are highly varied, even contradictory, but can be summarized with the following: (1) Regulated encouragement of introspection centered on experiences and memories, both traumatic and non-traumatic, pertaining to the intimacy of existence, to abolish or transmute these, seeking to modify the self-conception of personal history; (2) Implanting ideas, attitudes, events, experiences or ideologies different from those that the individual has and that characterize him/her; (3) Developing tests and measurement of modifications of brain images aimed at unmasking pretenses, falsehoods, lies or intentional inventions in trials or against litigants that seek to obtain direct advantages or indirect internal emotional retribution; (4) fixing responsibility and imputability of acts thanks to the findings of “brain prints” in neuroimages or of “test of guilty knowledge” in brain waves; (5) quantitative instrumental evaluation of the capacities to deliver informed consent that permits comparing them to objectively standardized tables that reveal whether an individual is incapable of making rational choices in her/his best interest because he/she cannot overcome the most minimal barrier; (6) prediction of future diseases by means of neuroimages that allows for taking measures pertinent to the person involved, loved ones, insurers and healthcare givers; (7) modifications of the personality with the aim of transforming and even recreating the self-identity of individuals with severe and disabling disorders or that have disturbing features, through ablation of parts of the brain or minimally invasive neuroengineering micro-implants that record, analyze and modify neuronal signals; (8) studies of cognitive and emotional functions like that of alertness, attention, memory, mood and sensations of wellbeing, pleasure and happiness through the use of drugs, a kind of “mind doping” with the aim of improving and perfecting the capacities and broadening its borders in both sick and healthy individuals [38–41].

It is clear that underlying these experiments, measurements with neuroimages and tests touch on central themes of existence which raise questions of the essence of our being and, with this, the legitimacy, scope and immediate and future effects of these assays. It should not be forgotten that science is not axiologically neutral but rather is guided by interests that, according to Habermas, are of domination or technical, practical or understanding of the sense and emancipatory or liberatory, and neuroethics is submitted to one or several of these to a greater or lesser degree [42, 43].

Without breaking them down, the most decisive dimensions to be modified can be listed: (1) Identity, recognition of itself as a unit despite the changes it has experienced and generated based on its own definition and that which others have provided; (2) privacy, an interior place that is experienced as inalienable and that no one can, should or has the faculty to transgress; (3) self-determination, the feeling of being the agent that decides actions and choices; (4) responsibility over ideas, acts and the direction of life, against which there must be ongoing accountability; (5) perfecting human dispositions and capacities evaluated according to ideals, goals, values and current ambitions.

## Neurocience of ethics

The most revolutionary, confrontational and controversial aspect is that neuroethics seeks to found ethics differently from the traditional philosophical and religious foundations. Without entering into the details, nuances and details that the theme requires for its precise articulation, we note that three aspects are central: human attributes that are involved in this study, research that deal with human freedom and the genesis of morality [44].

What questions does Neuroethics seek to answer, questions that have beset the West since its origins because they suppose a mode of understanding our way of being and determine the *tékhne tou biou*, the art of living, using the expression of Foucault? [45]. (1) the brain-body relationship, which began with the Greeks conceiving of reality as the *physis* and exploded violently with the metaphysical proposals of Descartes, proposals that meant the rupture with antiquity and the beginning of the modern age; (2) the identity of the self, essence of our interior self that provides us with a way of recognizing ourselves but can be reified if we do not differentiate between identity and selfness, identity-idem and identity-ipse according to Ricoeur, a difference that with difficulty allows for operationalizing and less quantifying [46]; (3) determinism-freedom, to feel the agent of one’s acts, thoughts and decisions although we know we are conditioned by nature itself, history and circumstance; (4) the essence of morality, that is, what is its primary consistency that seems to distinguish us from other living beings; y (5) a universal ethic, the possibility of a common morality for humanity beyond societies.

Are we truly free at the moment of deliberating and choosing our responses? Or is it our neuronal networks that determine our acts inadvertently although constantly? The bold essays of Libet, corroborated by Haggar and Eimer, raise doubts that the will is free to choose actions (understand one’s self by motives, know one’s self as agent and author, and believe one’ self to have the power to conduct one’s self distinctly in externally similar situations), it is rather an illusion that is projected subsequently into the past [47–50]. Measuring the cerebral potential of the secondary motor cortex it was determined that they are ahead of conscious decisions to make movements by 350 ms, which makes it plausible that there are extra-conscious neuronal mechanisms that effectively provoke supposedly willed actions that, in retrospect, are erroneously catalogued as products of will. Measurements of brain waves have shown equally illusionary the control and authority we suppose we have to execute thoughtless tasks, like moving a cursor or interfering with the alternate movements of fingers through magnetic transcranial stimulation [51]. Other neurobiologists adhere to this conception, because of which it is time to modify this belief sustained for centuries and fervently believed in by popular psychology, and accept that purported conscious causality, the affirmation that is chosen in daily life according to our will, is anything but real, is to attribute to us subsequently what is only the product of extrapersonal neuronal processes that occurred before [52].

If these essays demonstrate that “the function of the human brain is to take decisions” [53], does this imply that facing a moral dilemma the individual decides according to how his/her brain is structured neuronally and not wielding the best arguments? The hypothesis seems probable for two reasons: the ethical judgments are consecutive to extra-conscious neurochemical changes and, as Greene indicates, the functioning brain area corresponds to affective, sensate and intuitive, not to intellectual or rational areas [54]. Works from different centers agree in contesting the postulate of Kant: what we must do is determined by what we would do “if reason completely determined our will” [55].

Studies about moral dilemmas culminate in the possibly more surprising proposal: Is morality a product of the pact of living in society, or on the contrary, does its genesis lie in the evolution of the human mind since our appearance on earth? Of the numerous investigations dedicated to elucidating this matter, we choose two surveys responded to by volunteers, keeping in mind that these are rapidly replicated, refined and expanded in other centers. (1) A passenger on a train realizes that the conductor has fallen unconscious and is faced with a choice: if he does nothing six students walking on the rails will be hit by the train while if he presses a pedal the train will take a railway siding and hit a laborer working on the rails. Some 90 % of respondents say they would save the students. (2) A subject is in the way of a train out of control with six students on the track whose only escape is if the subject pushes aside an obese man beside him, which will cause the death of the obese man. Only 10 % of respondents find it legitimate to push the obese man out of the way even though the other six would die [56, 57]. The two dilemmas are similar in their structure, although they have opposite results. Scanner readings of brain waves while subject consider moral dilemmas like the situations above provide notable results; much more time is taken when it can be considered legitimate to harm another although it involves saving others and the reverse when it is appear that it is not legitimate, that is, in this case the decision is made more rapidly. In effect, faced with a personal ethical dilemma like directly hurting someone, it takes 7 s to decide what to do, while in an impersonal situation, that is to indirectly harm someone, it takes only 4 s. The response seems to be located in the different areas of the brain that are involved: in case of the personal dilemma, the frontal lobe, prefrontal medial cortex and limbic system and amygdala are activated, areas that are closely connected with emotions, affectivity and social cognition, while with the impersonal dilemma the areas involved are related to cognitive capacities.

Wilson has attempted to respond to this problem—personal and impersonal dilemmas—reviewing the results of research involving the evolutionary origin of social relationships [58]. Archeological studies have shown that in the Pleistocene primitive humans were hunter-gatherers, they lived in small communities of no more than 130 individuals of the same race and religion. The close, permanent and firm social link allowed them to survive and successfully confront the multiple dangers of an inhospitable and threatening environment. Life depended on mutual support, shared tasks and help provided among the members of the tribe. Thus a primary moral was born by means of a process of natural selection of affects, emotions and instincts that favored mutual help with those close by and systematically excluded or expelled strangers. Consequently, morality is the final expression of adaptation, a product of survival by natural selection. The brain imprints upon its neuronal circuits these codes of moral functioning with the passing of millions of years, and these codes, because they are recorded on neurons, synapses and circuits, are universal, extending throughout the human race. These rules of moral procedure are imposed rapidly on actions, without recourse to higher cognitive or intellectual rational processes, which would delay their action and lead to the demise of the subject and the tribe [56, 57, 59]. This morality of group salvation based on reciprocity to achieve personal salvation has to be ruled according to a categorical imperative that is very distinct what Kant proposed, which has become the code of modernity and that consists of something like “love the near and reject the distant”.

## Balance and perspective

Are we now as we used to understand ourselves before the era of neuroethics? Evidently not, and the change has altered how we conceive of ourselves morally, although still in the field of moral thought and not that of moral living. Do we have hard data to feel confident about our ethical inclinations? Yes and no, because as we do not believe in freedom without more precision, the known facts are ambiguous and at times contradictory, but offer hope of continuing in deepening our self-knowledge and in modifying our traditional way of judging ourselves. Can we take stock after such a short time? It may be risky and superficial, but the possibilities that are opened up are so important and profound that they deserve the following considerations, shallow as well as provisional and subject to ongoing review [60]. Three aspects are the most decisive: immediate advances and future perspectives, methodological problems involved and the ontology underlying the investigations.

1. The introduction of the empirical methods of the natural sciences has represented not only the contribution of concrete, effective and novel data to understand the moral phenomena but has also spectacularly broadened the field of investigation. But above all it represents a revolutionary perspective, and to a certain point, unprecedented in investigation. Although it has been a long held aspiration of western thought to construct an ethic based on belonging, firstly, necessarily and perhaps sufficiently, of the human being to the biological life of the universe, neuroethics has boldly made the leap. It has ascended from a reflective to a scientific level understood as possible of being replicated and refuted with arguments and standardized tests, or, employing a distorted version of the concept of Popper, falsifiabled [61]. It is only in its beginnings and the number of publications is growing exponentially so that it is difficult to predict the directions future scientific projects will take. Whatever the findings, they accrue and illuminate our current knowledge from unexpected visions. It is that knowledge, as Aristotle asserted, is born of wonder [62] and one cannot but be astonished by this approach and the avalanche of data from laboratories.
2. The methodological problems underlying investigations cannot be ignored. On the contrary, they should be examined in all their complexity to advance with safe passage and to avoid falling into old illusions as a result of haste, lack of rigorousness and enthusiastic but naïve conclusions by venturing into fields distant from one’s own specialty. They are multiple, with different scope, many with serious difficulties. A brief list indicates the basic questions: designs purposely developed for laboratories, that is far from reality and its complexities, giving priority to replicability over verisimilitude; the development of its findings seeking probabilistic before causal connections, perhaps because of the greatest difficulty of the latter; lack of confirmation of the analysis and results by third party researchers, although they attempt to respect the variables; frequent use of hypothesis, conceptions and models from very diverse disciplines, which lends itself to confusion, mistakes and lack of rigorousness because the data had not being acquired or processed in similar ways, that is, the extrapolation can fall into simplisticness; and abrupt ontological transit from brain waves to subjective internal experiences, without due precaution and training, as Varela noted in reference to the theme, although in a very different context [63].
3. Metaphysics, which is sustaining throughout the edifice, cannot be resolved by empirical science because science itself is the result of a certain ontological conception that makes it possible, and that, at least, comes from the modern epoch. As Zubiri said, modernity has consisted of a progressive entification of reality and a logification of thinking [64]. In turn, transcending the cerebral “is” to the moral “duty” is not a trival matter, as Hume emphasized terming it a naturalistic fallacy [65]. Heidegger pointed it out with special profundity, current natural science proceeds according to a special thought, calculating and re-presentative (*vor*-*stellende*) [66]. This means that its manner of understanding reality, in this case, the reality of human beings in their moral slope, is dominating, im-posing, target-ing, calculating, and with that, numbering. What gets the scientist thinking is not something little, their data are not minor, but a price must be paid: take the “objective” reality pertaining to our condition but without considering the “way of being” proper to manhood as such. Objective science sees morality as an object-posed (*ge*-*stezt*) before a subject, as a norm (*Ge*-*setzt*) that exists in the brain, moral consciousness as a reified court that investigates the self and its actions. It is overlooked that our being is nothing but a project-of-being, never defined for once and for all, free to choose us and reach ourselves, or lose ourselves and mimicking ourselves with things of everyday life. It is clear, although we choose at every moment, we are not free, that is, we do not have the freedom to give ourselves our first foundation since it has already been given, we are already-in(*schon*-*sein*)-the-world, we are thrown among things and persons from the moment we are born [67]. Neuroethics must not repeat the insufficiencies of the neuropsychological sciences born in laboratories of the early twentieth century, programs that were full of hope and ideals but ended in not fulfilling expectations because of lack of meditative thinking [68]. To transform medical ethics into scientific neuroethics requires reviewing its metaphysical basis to be at the height of its future conquests and thus avoid confusing, mixing and dissolving the data with that which allows and makes possible that the data appears [69, 70].
